# C-prior—implementation of the MAINTAIN instrument for patients with spinal pain: study protocol for a randomized clinical trial

**DOI:** 10.1186/s13063-026-09509-6

**Published:** 2026-02-10

**Authors:** Martha Funabashi, Garron Ives, Katherine A. Pohlman, Sheilah Hogg-Johnson, Murray Townsend, Luciana Macedo, Joyce Lee, Andreas Eklund

**Affiliations:** 1 Division of Research and Innovation, Canadian Memorial Chiropractic College, Toronto, ON Canada; 2 Department of Chiropractic, Université du Québec À Trois-Rivières, Trois-Rivières, QC Canada; 3 Research Center, Parker University, Dallas, TX USA; 4 Insight Health and Chiropractic, Private Practice, South West Rocks, NSW Australia; 5 Mount Forest Chiropractic, Private Practice, Mount Forest, ON Canada; 6 School of Rehabilitation Science, McMaster University, Hamilton, ON Canada; 7 The Institute of Environmental Medicine (IMM), KarolinskaInstitutet, Stockholm, Sweden

**Keywords:** Low back pain, Neck pain, Spinal pain, Chiropractic, Maintenance care, Manual therapy, Prevention, Randomized clinical trial

## Abstract

**Background:**

Spinal pain is a highly prevalent condition affecting a large part of the population. Chiropractic maintenance care (MC) is a management strategy intended to prevent spinal pain recurrent episodes and deterioration by treating patients at pre-planned intervals. A previous study showed that patients identified as a dysfunctional subgroup by the West Haven-Yale Multidimensional Pain Inventory (MPI) and receiving MC had fewer days with bothersome LBP. This suggests MC may have superior effectiveness in this subgroup of patients. The MPI, however, is not practical for daily clinical practice, prompting the development of the MAINTAIN instrument. This study aims to (1) assess the effectiveness and cost-effectiveness of stratified MC using the MAINTAIN instrument, and (2) assess the fidelity and procedure compliance of implementing the MAINTAIN instrument.

**Methods:**

This pragmatic randomized clinical trial will recruit 225 consecutive patients (18–65 years old) with significant (> 30 days in the past 12 months) recurrent spinal pain presenting to chiropractic clinics. After the initial 3 weeks (6 visits) of chiropractic care, patients will be randomized to receive either Stratified MC or Standard Chiropractic Care. Stratified MC: patients will complete the MAINTAIN instrument and be stratified to MC or symptom-guided care (clinicians’ judgment). Patients in the Standard Chiropractic Care arm will receive standard treatment based on the chiropractor’s judgment. The primary outcome is the total number of days with activity-limiting pain measured at 12 months. Secondary outcomes include the number of missed working days and loss of work productivity due to pain, pain intensity, disability, health-related quality of life, perceived improvement, and implementation of MAINTAIN instrument outcomes. An intention-to-treat protocol and generalized estimating equations (GEE) linear regression models will be used for analysis.

**Discussion:**

This study investigates the impact of using a clinical instrument to identify patients with recurrent spinal pain to target those who benefit most from a chiropractic MC approach. Strict inclusion criteria should ensure a suitable target group, and frequently collected repeated measures should provide accurate outcome assessment. The study is pragmatic and includes standard clinical procedures facilitating the generalizability and transferability of the results into clinical practice.

**Trial registration:**

ClinicalTrials.gov NCT05350254. Prospectively registered on April 22, 2022, last modified on December 08, 2023. The first patient was randomized into the study on December 21, 2023.

## Introduction

### Background and rationale

Spinal pain, consistingof neck, mid-back, and lower back pain, is a highly prevalent condition [1]. It is considered one of the largest economic health burdens in Western societies [1]. Specifically, low back pain is responsible for more years lived with disability than any other condition in the world, with more than 500 million cases in 2020 [2]. Neck pain affected 203 million people in 2020 [3]. The lifetime prevalence of thoracic pain is between 15.6 and 19.5% [4]. Despite the high overall disability burden for some people, including significant costs for individuals and health care systems, interventions aimed at secondary or tertiary prevention of spinal pain have not been widely evaluated. To date, the few efforts have been focused on the effects of exercise therapy with or without education in the prevention of recurrences or long-term disability [5].

Chiropractic maintenance care (MC) is a long-term management strategy introduced after an initial care plan is successful [6–17]. It is a type of secondary prevention and aims to prevent future episodes and deterioration of the condition by treating patients at pre-planned intervals, regardless of symptoms [6–17]. Chiropractic MC treatment usually includes regularly scheduled manual therapy, individual exercises, and lifestyle advice [18–23]. Historically, it has been reported that patients were mainly selected to receive MC based on their previous history of pain and on the effectiveness of the initial care plan [24–27].

Based on this, a pragmatic randomized clinical trial (RCT) investigated the effectiveness of MC in patients with recurrent and persistent low back pain [16]. Results presented a large variability [17] suggesting that there were sub-groups of patients with better outcomes (fewer days with pain and fewer visits) in the MC arm of the study.

Psychological [28, 29], behavioral [30], and social characteristics [31] of people with spinal pain are known to be important in the transition from acute to recurrent and persistent pain states [32–34]. The bio-psycho-social model is a framework that recognizes the influence of multiple interacting factors from different domains on spinal pain. Although not without limitations, it is the current leading framework guiding the management of spinal pain [31, 35–37]. Based on the cognitive-behavioral conceptualization of pain, the West Haven-Yale Multidimensional Pain Inventory (MPI) was developed to capture and measure the chronic pain experience [38]. The MPI instrument identifies three sub-groups [34] with similar characteristics within each sub-group. This classification has been shown to be reliable, valid, and useful in outcome-based research [39, 40]. The three different sub-groups are adaptive copers (AC; characterized by low pain severity, low interference with everyday life, low life distress, a high activity level and a high perception of life control), interpersonally distressed (ID; who tend to perceive negative responses by significant others to their pain behavior and complaints), and dysfunctional (DYS; characterized by high pain severity, marked interference with everyday life, high affective distress, low perception of life control and low activity levels) [38]. These sub-groups have been used in clinical settings to investigate neck and low back pain [41–43], temporomandibular disorders [44], headaches [45], fibromyalgia [46], and cancer pain [47] and have been associated with different clinical outcomes.

Secondary analyses of the data from the pragmatic RCT [48] revealed that patients in the DYS sub-group reported better outcomes from the MC approach. Specifically, these patients reported 30 fewer days with pain and an equal number of treatments compared to patients in the DYS sub-group who received the control (non-MC) intervention. Comparatively, patients in the ID and AC subgroup allocated to the MC treatment arm reported equal or more days with pain and 1.5 and 3.9 more treatments (respectively) compared to patients in the same MPI sub-group receiving the control intervention.

These results indicate that MC may be best recommended to patients in the DYS sub-group and not recommended to patients in the AC subgroup. Although there is evidence to support the validity and reliability of the MPI instrument, it was not designed to be used in daily clinical practice. Therefore, to apply the above-mentioned research results into clinical practice, the user-friendly MAINTAIN instrument was developed by the previous RCT investigators [49] based on the original MPI instrument.

The conclusions from a previous exploratory factor analysis by Turk and Melzack [50] were used to select which items would be most suitable for a short version of the original instrument. Different scoring algorithms were explored to find an appropriate model with an optimal compromise between diagnostic accuracy and ease of use in clinical settings. Ten questions from the original MPI questionnaire capturing 5 dimensions of the patient’s pain experience: pain severity, interference, life control, affective distress, and support were identified and combined into the MAINTAIN instrument [49]. It takes about 3 min in total for patients to complete and for clinicians to score. The MAINTAIN score ranges from − 12 to 48, and the proportion of a DYS sub-group profile increases with a higher score, whereas the proportion of an AC sub-group profile increases with a lower score.

Based on data collected from three different clinical populations [5] at a threshold of 18, the MAINTAIN instrument has a sensitivity of 95.8% and specificity of 64.3% for the DYS sub-group profile [5, 12, 16]. At a threshold of 22, the MAINTAIN instrument has a sensitivity of 81.1% and a specificity of 79.2% for a positive test for the DYS sub-group profile. Based on these statistics, three categories have been suggested based on MAINTAIN scores: − 12–17 = not a candidate for MC, 18–21 = good candidate for MC, and 22–48 = very good candidate for MC [49].

Despite these encouraging results, the usability and impact of the MAINTAIN instrument applied in clinical practice remain unknown. Therefore, it is fundamental to investigate the outcomes and process of implementing the MAINTAIN instrument into clinical practice.

### Objectives

The overall goal of this randomized clinical trial is to investigate the impact and process of implementing the MAINTAIN instrument in clinical practice to classify patients with spinal pain. The primary aim of this study is to assess the effectiveness and cost-effectiveness of stratified MC guided by the MAINTAIN instrument score compared to standard chiropractic care (without the MAINTAIN instrument). The secondary aim is to assess the fidelity and procedure compliance of implementing the MAINTAIN instrument.

We hypothesize that participants receiving the stratified intervention guided by the MAINTAIN instrument will present better outcomes (e.g., fewer days with activity-limiting pain and lower pain intensity and disability) at 12-months compared to the standard chiropractic care intervention. It is also hypothesized that compliance with procedures to implement the MAINTAIN instrument will be high.

### Trial design

This is a type 1 hybrid effectiveness-implementation study.

## Methods: participants, interventions, and outcomes

### Study setting

Community-based chiropractors located anywhere in Canada will be invited to participate in this study through social media and advertisements shared with members from their professional provincial organizations. All advertisements will have approval by the research ethical board. All participating chiropractors will be trained in patient recruitment and study protocol with pre-recorded videos and a mandatory live webinar training session. The research coordinator will contact participating chiropractors monthly to ensure protocol adherence, participant engagement, and opportunities for potential troubleshooting.

### Eligibility criteria

Inclusion and exclusion criteria for patients to participate in this study are presented in Table 1.

**Table 1:** Eligibility criteria

Inclusion criteria	Exclusion criteria
• Age 18–65 years • Neck pain, mid-back pain, and/or low back pain with or without arm or leg pain for more than 30 days during the past year • Previous spinal pain episodes • No chiropractic care in the past 3 months • Access to a mobile phone and email • Ability to send and receive SMS (text messages)	• Pregnancy • Serious pathology (i.e., acute trauma, cancer, infection, cauda equina syndrome, osteoporosis, vertebral fractures) • Contraindications to manual therapy • Patients with specific spine conditions, such as radiculopathy and spinal stenosis

These criteria will be assessed at the first visit self-reported by patients (all inclusion criteria and pregnancy status) and by clinicians (serious pathology, specific conditions, and contraindications to manual therapy). Final eligibility will be determined by clinicians during the first visit, prior to randomization.

### Study procedure

**Fig. 1:**
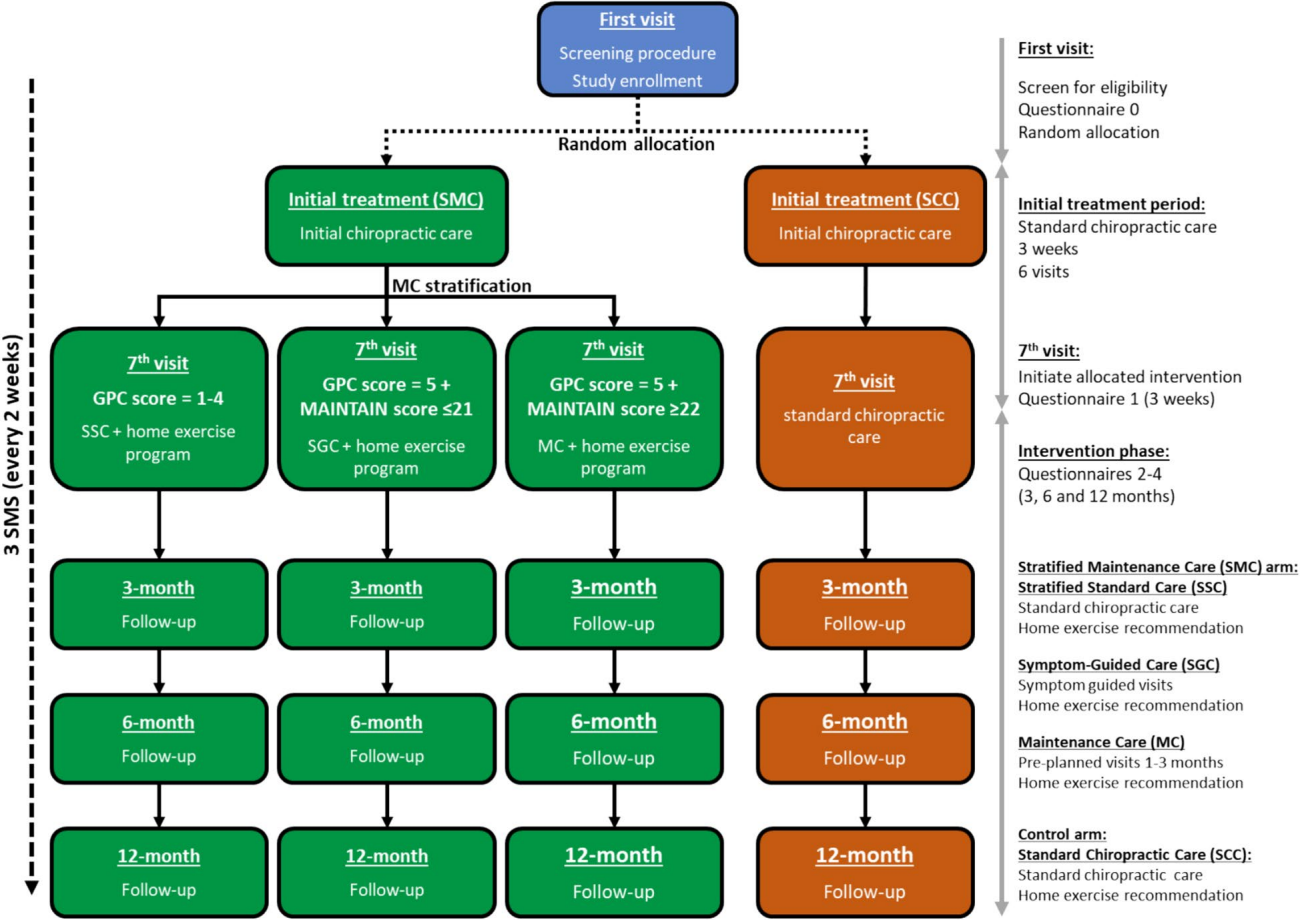
Study flow and intervention arms

The general flow of this trial is shown in Fig. 1. At each participating chiropractic office, consecutive patients that meet the eligibility criteria (Table 1) will be invited to participate in the study by the office’s front desk or by the chiropractor (whatever works best for their office) when they call to book their appointment. The invitation of consecutive patients was reinforced in the mandatory webinar training. Eligible patients who agree to participate in the study will be asked to arrive at the office for their first chiropractic and study visit about 30 min before their appointment to get details about the study via an introductory video and study information letter and to sign an informed consent electronically. Patients not meeting the eligibility criteria or choosing not to participate in the study will have no change in the care provided by their chiropractor.

At their first visit prior to their appointment, participating patients will complete the first electronic questionnaire in REDCap (Questionnaire 0-patient), which includes the MAINTAIN instrument. Once patients complete Questionnaire 0-patient, their chiropractor will examine patients to determine final eligibility and complete Questionnaire 0-clinician also in REDCap.

### Randomization

Participants will be randomly allocated to one of the two intervention arms at the first visit, as part of Questionnaire 0-clinician (immediately after eligibility criteria confirmation): Stratified Maintenance Care (SMC) or Standard Chiropractic Care (SCC), with the number of individuals equally balanced between groups. Central randomization will be performed, using block randomization stratified by clinician, performed by the study biostatistician (SHJ) using SAS software. To ensure the allocation sequence is concealed from the researchers and participating clinics, varying block sizes of 2, 4, and 6 will be used. The unique randomization orders will be uploaded into REDCap (Research Electronic Data Capture system, Vanderbilt University, Tennessee) [51]. Each chiropractor will indicate, in their Questionnaire 0-clinician, the number of the patient (i.e., first patient enrolled = #1; second patient enrolled = #2, etc.), which will reveal that patient's group allocation. Importantly, chiropractors will only have access to the MAINTAIN instrument score for patients allocated to the MC group.

### Blinding

The randomization will be blinded to the study team in order not to influence clinicians during the training webinar and other communications during the enrollment process. Participating clinicians will be unaware of the individual allocation of their patient until they have indicated the patient number on REDCap after consent is given and eligibility is confirmed. Due to the nature of chiropractic care, study participants (including clinicians and patients) cannot be blinded to the treatment.

### Sample size

There will be one primary comparison between the two study arms (SMC vs SCC) and a secondary comparison between those classified as DYS across study arms, anticipating that approximately half of each study arm will be classified as DYS. Based on the previous study [17], to detect a difference in the number of days with pain over 12 months [17] between the two main study arms corresponding to a medium Cohen’s effect size of 0.5, with an α-level of 0.05, power of 0.80, a sample consisting of 90 patients per arm would be required. Given that our study includes 2 arms and to account for potential dropouts (20%), our goal will be to recruit 220 patients [52]. Therefore, 15 clinicians will be recruited to collect data from 15 patients each.

### Data collection

The automated SMS track, via Twilio System, will be used to collect SMS, and REDCap will be used to collect electronic questionnaire data. Each participating clinician will complete an initial questionnaire (Questionnaire 0-clinician) to record their individual characteristics.

Upon Questionnaire 0-patient completion, participating patients will be automatically registered in the Twilio system, given a confidential study ID linked to the clinician from which they are receiving treatment, and automatically scheduled to receive 3 questions via SMS every 2 weeks for 12 months. The follow-up questionnaires (Questionnaire 1–4) will be automatically sent via e-mail to both participating patients and clinicians at 3 weeks, 3, 6, and 12 months, respectively.

Data collection will be monitored daily in real-time, and contact with participating patients and clinicians will ensure high response rates. Specifically, in case of email delivery notification from REDCap or identification of incomplete questionnaires, email reminders will be sent to participating patients and clinicians.

## Interventions

The intervention this study will investigate is the use of the MAINTAIN instrument to guide the classification of patients and stratification of the MC approach. Specifically, the MAINTAIN instrument score will be used in combination with patients’ response to initial chiropractic care to determine their stratification within a tailored MC approach. This stratified strategy (Stratified MC) is based on prior investigations observing that both clinical response and psychosocial profiles contribute to MC outcomes [17, 44, 45].

### Initial care

As shown in Fig. 1, this trial will comprise two arms, of which all participating patients will receive pragmatic chiropractic care for the initial 3 weeks (6 visits) with interventions tailored to each patient based on clinicians’ judgment. After the initial treatment period, each patient will initiate the allocated intervention: Stratified MC (intervention arm) or Standard Chiropractic Care (control arm).

#### Stratified MC—intervention arm

The Stratified MC group will be first classified by their response to the initial 6 visits using the global perceived change score (GPC) collected at their 3-week (Questionnaire 1): *responding* (GPC score = 5) or *not responding* to care (GPC score ≤ 4). Based on their GPC score, participating patients will be further stratified based on their MAINTAIN score. A MAINTAIN score ≤ 21 is classified as *not a candidate for MC*, and a MAINTAIN score of ≥ 22 is classified as a *very good candidate for MC*. A combination of both the GPC and MAINTAIN scores will determine the stratification to one of the following groups:

- *Stratified Chiropractic Care (SCC):* Participating patients classified as *not responding* (GPC score ≤ 4) to the initial 3 weeks of care will be offered an alternative treatment based on clinicians’ judgment, which will be recorded. Home exercises will be recommended.
- *Symptom Guided Care (SGC):* Participating patients classified as *responding* (GPC score = 5) to the initial 3 weeks of care and *not a candidate for MC* (MAINTAIN score ≤ 21) will return for further interventions and manual treatment if they have a relapse or exacerbation of symptoms. Home exercises will be recommended.
- *Maintenance Care (MC):* Participating patients classified as *responding* (GPC score = 5) to the initial 3 weeks of care and *very good candidate for MC* (MAINTAIN score ≥ 22) will be recommended MC with pre-planned visits at 4–12-week intervals (aiming at increasing the interval as soon as possible), including tapering manual treatments and home exercise recommendations. Participants in this group can return to the clinic in between their pre-scheduled visits in case they experience a flare-up. The rationale behind choosing only the higher category for MC (MAINTAIN score ≥ 22) in this trial is related to the instrument’s sensitivity and specificity. Within the middle category, “MAINTAIN score 18–21,” the uncertainty is higher for misclassification of the psychological profile (AC vs. DYS), and a more shared decision-making process is required in clinical practice to determine the most suitable treatment strategy. This does not align well with the design of this randomized trial, where one group must follow the assigned intervention. Therefore, the higher cutoff was used to ensure that the correct patients receive the most appropriate treatment strategy in the intervention group, thereby minimizing the risk of AC receiving MC.

#### Standard chiropractic care (SCC)—control arm

Participating patients will receive a SCC in which treatment will be provided based on clinicians’ judgment and home exercise recommendations. This may or may not include MC depending on clinicians’ standard operating procedures. All treatments provided will be recorded.

#### All groups

Initial care will include home exercise recommendations with progression tailored to each participant based on their initial assessment. In addition, participants will be encouraged to increase physical activity based on their preferences and functional limitations (i.e., walking program, bike, swimming). Participants will be instructed to increase activity using graded activity principles [53]. All patients will be given an infographic with graded activity guidelines (e.g., pacing, quotas). In addition, all groups will receive interventions based on clinicians’ judgment and reassuring messages focusing on developing self-efficacy while encouraging the minimization of kinesiophobia, catastrophizing, and passive coping behaviors, in accordance with contemporary pain science.

Once completed participation, a purposeful sample of chiropractors and patients from both groups will be invited for semi-structured qualitative interviews to explore their experience of being part of the study to get a further understanding of barriers and facilitators for using the MAINTAIN instrument. A semi-structured interview guide will be developed a priori. One-to-one interviews will be conducted by one of the investigators using a virtual platform (Zoom Video Communications, Inc., CA, USA). The session will be recorded, transcribed verbatim. We will aim to recruit a convenient sample of 12–15 participants per group (chiropractors and patients) that completed the study intervention to be interviewed.

### Concomitant care

Given that this is a pragmatic study, the concomitant use of other treatments and interventions will not be restricted during the study. The intervention will not interfere with other regular medical care and medications, which will continue as usual during the study.

### Outcome measures

Our primary outcome measure is the number of days with activity-limiting pain (recorded via SMS) measured bi-weekly over 12 months. Secondary outcome measures include missed working days due to pain, loss of work productivity due to pain, pain intensity, global perceived change, self-reported activity limitation, self-rated health, and pain self-efficacy. Table 2 shows all outcome measures recorded in the SMS and electronic questionnaires.

**Table 2:** Summary of outcome measures

Time point	Questionnaire	Patient outcome measures	Clinician outcome measure
Every 2 weeks	SMS	- Total number of days with activity limiting pain in the past 2 weeks - Total number of missed workdays due to pain in the past 2 weeks - Experienced loss of productivity at work due to pain in the past 2 weeks	
First visit (baseline)	0	- Demographic data - Inclusion criteria - Pain intensity (NRS) [54] - Disability (Neck Disability Index [55]/Roland-Morris Disability Questionnaire [56]) - MAINTAIN instrument [49] - Health-related QOL (EQ-5D-5L) [58] - Self-efficacy (Pain Self-Efficacy) [59] - Physical Activity (IPAQ) [60] - Expectations	- Clinical impression - Implementation data
3 weeks	1	- Global Perceived Change [61] - Adverse events - Pain intensity (NRS) - Disability (Neck Disability Index or Roland-Morris Disability Questionnaire) -MAINTAIN instrument - Health-related QOL (EQ-5D-5L) - Self-efficacy (Pain Self-Efficacy) - Physical Activity (IPAQ) - Expectations	- Implementation data - Clinical impression - Date of visits and content (since last follow-up)
3 months	2		
6 months	3		
12 months	4		

### Baseline and follow-up assessments

A summary of baseline and follow-up assessments is detailed in Table 2. Baseline data (Questionnaire 0) will be collected from both clinicians and patients at the first visit. Clinician data will include implementation data as well as their clinical impression of the patient. Patient data will be collected on demographics, current pain (numeric pain scale (NRS) [54]), pain in the past week (NRS), activity limitation/disability (neck disability index (NDI) [55]/Roland-Morris Disability Questionnaire (RMDQ) [56]), psychological and behavioral consequences of pain (MPI [57]), self-rated health (EQ-5D [58]), the MAINTAIN instrument [49], beliefs of patients on their ability to function with pain (pain self-efficacy questionnaire [59]), physical activity (International Physical Activity Questionnaire (IPAQ) [60]), and expectations of treatment.

**Fig. 2:**
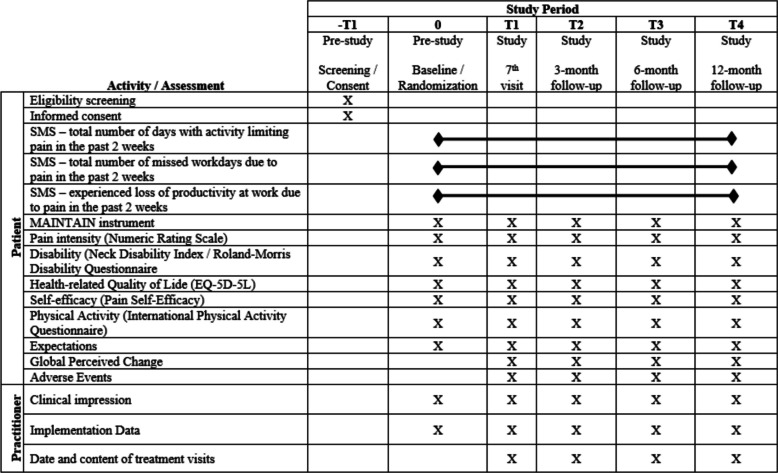
Schedule of participant enrollment, interventions, and assessments

Follow-up assessment (Questionnaire 1–4) will be completed at 3 weeks, 3 months, 6 months, and 12 months, also collecting data from clinicians and patients. Practitioners will provide data on implementation and clinical impression of the patient, as well as expectations of care if the patient is receiving MC (Yes/No), and the date and content of treatment visits. In addition to these questionnaires, patients will also receive an SMS every 2 weeks to collect data on activities limited due to pain in the last 0–14 days, missed working days due to pain, and loss of work productivity due to pain. Figure 2 presents the schedule of participating practitioners’ and patients’ enrollment, interventions, and assessments.

### Safety monitoring

A Data and Safety Monitoring Board (DSMB) will monitor participant safety, data quality, and the progress of the trial. The DSMB includes a physiotherapist with expertise in patient safety and two chiropractors: one with expertise in clinical trials and one with expertise in patient safety. The DSMB will communicate frequently via email throughout the study, formally meet every 6 months, and monitor data quality and adverse events. While there is no pre-planned auditing of the trial, the institutional research ethics board may perform post-approval monitoring and auditing of the trial conduct.

Based on the literature, for both groups, we anticipate only mild adverse events (AEs) commonly reported following chiropractic treatments, such as mild discomfort/pain and stiffness [62, 63]. All patient follow-up questionnaires will include specific questions about AEs. If an AE is reported, the principal investigators will be notified immediately via email for documentation and reporting in the final manuscript. Upon notification, at least one of the principal investigators will review the AE report by accessing the participant’s response on REDCap and identify the type of AE and severity based on pre-defined criteria [64]. If any serious adverse event (SAE) that interferes with study protocol compliance is identified, it will be reported to the ethics board, the respective chiropractor will be notified, and patients will be instructed to discontinue the study.

### Provisions for post-trial care

As no adverse events beyond normal transient mild discomfort/pain are expected, no follow-up plans will be included in this study.

### Protocol amendments

Any modifications to the protocol which may impact the conduct of the trial, potential benefit of patients or may affect patient safety, including changes of study objectives, trial design, participant population, sample sizes, trial procedures, statistical analyses, or significant administrative aspects will require a formal amendment to the protocol. Such amendments will be agreed upon by the research team and, if needed, approved by the local Research Ethics Board. Administrative changes of the protocol (e.g., minor corrections and/or clarifications) that have no effect on how the trial is conducted will be agreed upon by the research team and documented.

### Project timeline

Recruitment will run from November 2023 to May 2026. Data collection is expected to be completed in July 2027, with data analysis and synthesis conducted in the fall of 2027.

### Data analysis plan

#### Primary outcome

An intention-to-treat protocol will be used, and estimates will be reported with arithmetic means and 95% confidence intervals. The primary outcome (number of days with activity-limiting pain) will be analyzed similar to Eklund et al. (2018,2019) [17, 48]. The number of days with activity-limiting pain will be modeled using generalized estimating equations (GEE) linear regression, using appropriate correlation structure and robust variance estimator. Terms for study arm, time, and study arm by time interaction will be included. A term for MC candidate (yes or no) will be added to this model to investigate stratification and its relationship to outcomes further.

Following a previous study that conducted a similar analysis [16], cost-effectiveness will be investigated both from the health care and societal perspectives. In the health care perspective only costs directly associated with the intervention will be included whereas in the societal perspective all available costs, direct and indirect modeled production loss costs, will be used. Direct medical costs considered in the study are treatment costs (treatment fee) and time loss during treatment and travel (an average time for travel is estimated and added to an average patient visit time). Indirect costs, such as production loss, will be estimated using the human capital approach, where lost time is valued using the hourly wage [65]. Since the data collection is performed between 2023 and 2025, all costs will be adjusted for inflation using a 3 to 5% inflation rate, with the baseline year of the study as a base year [66] and calculated in CAD$. Intervention effectiveness will be estimated by the primary outcome “number of days with a bothersome spinal pain” and self-rated health (EQ5D) as a secondary outcome. The results will be reported as cost-effectiveness planes, cost acceptability curves, and incremental cost-effectiveness ratios (ICER) where the extra cost per extra unit of health effect will be estimated for days with bothersome pain and quality-adjusted life years (QALYs). No interim analyses are planned for this study.

#### Secondary outcomes

The secondary outcomes will be analyzed and reported independently of the primary outcome. Missed workdays due to pain, loss of work productivity due to pain, pain intensity, global perceived change, self-reported activity limitation, self-rated health, and pain self-efficacy will be reported with arithmetic means and 95% confidence intervals, and GEE linear regression models will be used.

Qualitative interviews data will be analyzed using interpretive description. An inductive approach to thematic analysis will be used to facilitate coding of the data driven by the responses rather than preconceived theoretical or analytical knowledge. Transcripts will be coded, and key themes will be identified. Several well-established techniques to ensure methodological rigor and trustworthiness for interpretive description will be used, including member checking (participants’ comments on any developed themes), verification (researchers converge on recognizing “identical patterns” in the data), referential adequacy (providing enough quotes to ensure the findings fit the data), maintaining an audit trail (records of decisions made), and maintaining a reflexive journal (to help the interviewers identify how their experience may influence the study results).

The RE-AIM framework will be used to assess the impact and implementation of the MAINTAIN instrument using other qualitative and quantitative analyses, see Appendix 1 summary table.

### Ethical considerations

This study has ethics approval from the Canadian Memorial Chiropractic College’s Research Ethics Board (Approval #: 2203B02). Written informed consent will be obtained from all participants prior to study entry. The informed consent document includes information regarding the potential use of de-identified data beyond the specific objectives of this study.

### Dissemination plans

The results from this trial will be disseminated regardless of the magnitude or direction of effect. Results from this trial will be submitted for publication in peer-reviewed journals and presented at national and international conferences related to chiropractic care (e.g., World Federation of Chiropractic Research Conference). Peer-review publications will be reported following CONSORT guidelines (including CONSORT-Harms extension) and TIDieR checklist. An infographic summary of our results will be developed and disseminated to all Canadian provincial chiropractic associations to be distributed to their respective members. This summary will also be distributed to students, faculty, and staff of the investigators’ institutions. Trial participants who are interested in receiving a summary of study results will also receive the study infographic via mail.

## Discussion

Although spinal pain has been a leading cause of disability and a healthcare challenge for many years, effective preventive strategies for recurrent episodes and deterioration for those who need it remain scarce. Previous trials found that people with persistent and recurrent low back pain in the Dysfunctional sub-group (based on the MPI) report better outcomes when receiving a MC approach [17, 48]. However, the MPI was not designed to be used in clinical practice, which led to the development of the MAINTAIN instrument specifically to identify patients who would most benefit from a MC approach in clinical practice [49].

A key benefit of having a brief instrument, such as the MAINTAIN instrument, is that it can be employed during the clinical encounter. The simple scoring algorithm allows clinicians to immediately use its results, providing an overview of the psychological aspects of patients’ pain experience. This can also inform clinicians’ follow-up questions targeted to specific domains. Additionally, MAINTAIN instrument scores can be used to help establish treatment goals tailored to each patient’s needs and preferences, thereby better aligning the management plan with a patient-centered approach.

If this trial is successful, the MAINTAIN instrument may contribute to reducing healthcare costs by identifying patients who would benefit from a MC approach, leading to more effective management of these patients. Spinal pain significantly impacts individuals, the healthcare system, and society [2–4], resulting in diminished quality of life, increased disability, lost productivity, and substantial healthcare expenses. Therefore, developing and implementing effective preventive strategies for targeted populations is imperative as it can significantly improve individual health, well-being, and quality of life.

By using a pragmatic design, the results of this trial will reflect real-world clinical practice. Additionally, the findings can be readily translated into practice and will provide a comprehensive understanding of the barriers and facilitators associated with the implementation of the MAINTAIN instrument in clinical practice. This, in turn, will be highly relevant to assessing whether this instrument should be recommended and implemented in clinical practice.

## Trial status

Protocol version 1.0. The trial is ongoing, and participants (chiropractors and patients) are being recruited. Study recruitment started in November 2023 and is anticipated to continue until May 2026. Data collection is expected to be completed in July 2027.

## Data Availability

Primary analysis of data will be conducted by the team biostatistician (SHJ). Other members of the research team will also have access to de-identified data to perform the analysis of secondary outcomes, such as qualitative interviews and implementation assessment. Access to the participant-level dataset will be based on the established collaboration agreements including data transfer agreements.

## Appendix 1

**Table 3:** Summary of outcomes, measures, and analysis plan using the RE-AIM framework

RE-AIM element	Outcome measure; source	Data collection time point; data analysis
**Reach** *The reach of the intervention into the target population*		
Interest	Number of participants enrolled in the study across all sites (chiropractic clinics) until last participating patient completes follow-up; study records	End of last follow-up; descriptive statistics
Recruitment	Proportion of eligible patients who consent and enroll compared to those that are invited but decline to participate	End of recruitment; proportions
Use of qualitative methods to understand reach/recruitment	Qualitative interview with chiropractors and patients	Qualitative description
**Effectiveness** *Positive and adverse effects of the intervention*		
Measure of primary outcomes	number of days with activity limiting pain; self-report	Bi-weekly; GEE linear regression
Measure of secondary outcomes	Self report: Pain intensity (NRS) Disability (NDI/RMDQ) Health related QOL (EQ-5D-5L) Self-efficacy (pain self-efficacy) Physical activity (IPAQ)	T_0_, T_1_ T_2_, T_3_, T_4_; GEE linear regression
Effectiveness perspectives	Qualitative interviews with patients and chiropractors	End of study participation: interpretative description
**Adoption** *Representation of setting and intervention agents who are willing to initiate and actively participate in program*		
Setting level		
Number of sites involved in the study	Number of chiropractors and clinics participating	T_4_; descriptive statistics
Characteristics of chiropractors	Characteristics of chiropractors	T_4_, descriptive statistics
**Implementation** *Fidelity to the intervention and adaptions*		
Assessment of patient’s symptoms	Implementation questionnaire: “Did you consider if the patient had more or less than 30 days with pain during the past 12 months? Yes/No” Implementation questionnaire: “Did you consider the patient’s GPE? Yes/No”	T_1_ T_2_, T_3_, T_4_; descriptive statistics
Use of MAINTAIN instrument	Implementation questionnaire: Did you consider the MAINTAIN score?	T_1_ T_2_, T_3_, T_4_; descriptive statistics
Assessment of progress towards the goals set in the treatment plan	Implementation questionnaire: Did you assess progress towards the patient-specific functional goals set during the previous visits?	T_1_ T_2_, T_3_, T_4_; descriptive statistics
Exercise prescription	Implementation questionnaire: Did you give the Graded Activity plan to the patient?	T_1_; descriptive statistics
Exercise compliance	Implementation questionnaire: Did you assess exercise compliance? Yes/No	T_2_, T_3_, T_4_; descriptive statistics
Number of sessions	Questionnaires: number of sessions attended per participant Number of maintenance care appointments per group Number of symptom-based care appointments per group	T_4_; descriptive statistics T_4_; descriptive statistics, negative binomial regression T_4_; descriptive statistics, negative binomial regression
Practice change	Qualitative interviews with chiropractors and patients	End of study participation: Interpretative description
**Maintenance** *Extent to which program becomes sustained over time*		
Practice change	Qualitative interviews with chiropractors	End of study participation: Interpretative description
